# Incidence, Clinical Outcome and Risk Factors of Intensive Care Unit Infections in the Lagos University Teaching Hospital (LUTH), Lagos, Nigeria

**DOI:** 10.1371/journal.pone.0165242

**Published:** 2016-10-24

**Authors:** Anthony A. Iwuafor, Folasade T. Ogunsola, Rita O. Oladele, Oyin O. Oduyebo, Ibironke Desalu, Chukwudi C. Egwuatu, Agwu U. Nnachi, Comfort N. Akujobi, Ita O. Ita, Godwin I. Ogban

**Affiliations:** 1 Department of Medical Microbiology and Parasitology, College of Medical Sciences, University of Calabar, Calabar, Nigeria; 2 Department of Medical Microbiology and Parasitology, College of Medicine, University of Lagos, Nigeria; 3 Department of Anaesthesia, College of Medicine, University of Lagos, Nigeria; 4 Department of Medical Microbiology and Parasitology, Faculty of Medicine, Nnamdi Azikiwe University, Nnewi Campus, Awka, Nigeria; 5 Department of Immunology, Faculty of Medicine, Nnamdi Azikiwe University, Nnewi Campus, Awka, Nigeria; Azienda Ospedaliero Universitaria Careggi, ITALY

## Abstract

**Background:**

Infections are common complications in critically ill patients with associated significant morbidity and mortality.

**Aim:**

This study determined the prevalence, risk factors, clinical outcome and microbiological profile of hospital-acquired infections in the intensive care unit of a Nigerian tertiary hospital.

**Materials and Methods:**

This was a prospective cohort study, patients were recruited and followed up between September 2011 and July 2012 until they were either discharged from the ICU or died. Antimicrobial susceptibility testing of isolates was done using CLSI guidelines.

**Results:**

Seventy-one patients were recruited with a 45% healthcare associated infection rate representing an incidence rate of 79/1000 patient-days in the intensive care unit. Bloodstream infections (BSI) 49.0% (22/71) and urinary tract infections (UTI) 35.6% (16/71) were the most common infections with incidence rates of 162.9/1000 patient-days and 161.6/1000 patient-days respectively. *Staphylococcus aureus* was the most common cause of BSIs, responsible for 18.2% of cases, while Candida spp. was the commonest cause of urinary tract infections, contributing 25.0% of cases. Eighty percent (8/10) of the Staphylococcus isolates were methicillin-resistant. Gram-negative multidrug bacteria accounted for 57.1% of organisms isolated though they were not ESBL-producing. Use of antibiotics (OR = 2.98; p = 0.03) and surgery (OR = 3.15, p< 0.05) in the month preceding ICU admission as well as urethral catheterization (OR = 5.38; p<0.05) and endotracheal intubation (OR = 5.78; p< 0.05) were risk factors for infection.

**Conclusion:**

Our findings demonstrate that healthcare associated infections is a significant risk factor for ICU-mortality and morbidity even after adjusting for APACHE II score.

## Introduction

The prevalence of ICU-acquired infections is significantly higher in developing countries than in industrialised countries, varying between 4.4% and 88.9% [1]. A recently published World Health Organisation (WHO) review revealed that “In low- and middle-income countries the frequency of ICU-acquired infection is at least 2–3 higher than in high-income countries; device-associated infection densities are up to 13 times higher than in the USA” [2]. While critical care medicine is a science in the developed world, it is just emerging in Nigeria with a population of over 170 million people. Only 10 of 36 states of the federation have ICU beds. A study conducted in Jos, Nigeria has estimated the prevalence of nosocomial infections is approximately 6% and a disproportionate 20% of these occur in critically ill patients in intensive care units [3]. Another study from Northern Nigeria demonstrated 37 (30.8%) positive bacterial isolates from samples collected from ICU patients [4].

A recent European multicentre study posted that the proportion of infected patients in intensive care units can be as high as 51%; most of these are health care associated [2]. Endotracheal intubation with mechanical ventilation increases the risk of nosocomial pneumonia by 6 to 21 times [5]. Central venous catheterization accounts for 97% of all nosocomial blood stream infection [6]. Urinary catheterization is the most important risk factor for acquisition of nosocomial urinary tract infections. Nasotracheal intubation is the most significant risk factor for acquisition of nosocomial sinusitis [7]. Other established risk factors include co-morbidities.

Critically ill patients with severe sepsis in intensive care units (ICUs) require lengthy and expensive management, with an associated high mortality, with rates ranging from 30% to 50% [8]. ICU-acquired infections has been found to be an independent risk factor for ICU-mortality even after adjusting for possible co-morbidities [9,10,11]. Strategies to reduce these rates is dependent on accurate and adequate data however, there is paucity of local data on ICU-acquired infections in our setting, thus, there is an overdependence on data from other climes which do not necessarily reflect the local realities. Also, health care-associated infections (HAI) are known to vary in terms of aetiology, resistance pattern and risk factors even in different units in the same hospital setting.

To initiate necessary policies that are critical to effective treatment of ICU-acquired infections and prevent antibiotic resistance development, there should be surveillance of bacterial aetiologies and infection patterns. This study determined the prevalence, risk factors, clinical outcome and microbiological profile of hospital-acquired infections in the intensive care unit of a Nigerian tertiary hospital.

## Methods

This was a prospective and observational study. It was conducted in the ICU of the Lagos University Teaching Hospital, Nigeria (LUTH), from September, 2011 to July, 2012. LUTH is a 761 bed tertiary hospital with a six-bed ICU that admits critically ill patients from all specialties. The ICU admits approximately 220 patients annually. This study was approved by the Health Research and Ethics Committee of the Lagos University Teaching Hospital, Lagos (S2 File).

All patients that were fifteen years of age and above whose surrogates gave informed written consent (S3 File) over the study period were recruited into the study. Exclusion criteria were those patients whose anticipated stay in the ICU would be less than 48 hours or those unwilling or whose surrogates did not give consent. All written consent forms were stored in a locked filing cabinet as approved by the ethics committee. A structured proforma (S1 File) was used to collect patients’ relevant information. The proforma was divided into 4 sections: socio-demographic, medical and drug history, possible risk factors, and outcome. Severity of underlying diseases was assessed within 24 hours of admission using the Acute Physiological and Chronic Health Evaluation index (APACHE II score) (S4 File) [12]. A score of twenty and above was taken as a severe premorbid state where the patient has about 40.0% probability of dying. The endpoint of the study was patient discharge from the ICU or death.

### Definitions

Health-care associated infections (HAIs) are defined as an infection developing >48hours after hospital admission or within 30 days after discharge from a hospital [13].

Patient-days is the total number of days that patients were in the ICU during the period of study.

Multi-drug resistance (MDR) was defined only for gram-negative bacteria, as resistance to three or more groups of antibiotics [14].

### Sample collection and processing

Urine, blood, endotracheal aspirate, cerebrospinal fluid, wound aspirate and stool samples were aseptically collected from 71 recruited patients on the first day of admission into the ICU (no bronchoalveolar lavage specimen was collected). Repeat samples were taken after 48 hours of admission. Further samples were collected whenever there was clinical suspicion of infection; otherwise, they were collected weekly. All samples were transported to the clinical microbiology laboratory for immediate processing. Blood was cultured in the BACTEC culture system 9050 (Becton Dickinson, New Jersey, US). The other samples were processed according to established standardized protocol. Anaerobic cultures were not done. Isolates were identified to the species level using MicroBact^®^ (Oxoid, UK). Quality control was done using *E*. *coli* (ATCC 25922) and *S*. *aureus* (ATCC 25923).

### Antimicrobial susceptibility testing

Antimicrobial susceptibility testing was determined by Kirby-Bauer disc diffusion method [15] on Mueller-Hinton agar (MHA) plates according to CLSI guidelines [16]. The tests were controlled with *E*. *coli* (ATCC 25922), *S*. *aureus* (ATCC 25923), *P*. *aeruginosa* (ATCC 27853). All Staphylococci were screened, according to Clinical Laboratory Standards Institute guideline [16]. An isolate was described as methicillin-resistant Staphylococcus aureus (MRSA) or methicillin-resistant coagulase-negative Staphylococcus (MRCoNS) if the zone of inhibition was ≤22mm around a cefoxitin(30μg) disc. The positive control strain was *S*. *aureus* (ATCC 25923) for positive control.

#### Antimicrobial resistance testing

The presence of ESBLs was suspected if an isolate of K. pneumoniae or E. coli demonstrated resistance to one or more of the indicator beta-lactam antibiotics–ceftriaxone, cefotaxime or cefepime [17]. The screening and confirmation of ESBLs production by the gram negative bacteria E. coli and Klebsiella spp. was carried out using CLSI criteria [16]. For the confirmatory test (phenotypic), a double disc diffusion synergy test was performed. An extended zone of inhibition toward the disc containing clavulanic acid (dumb-bell shape) was interpreted as synergy, indicating the presence of an ESBL. The quality control strain used was E. coli (ATCC 25922).

All Staphylococcus spp. isolated were subjected to testing which was performed according to the CLSI guidelines [16]; 15μg erythromycin and 2μg clindamycin discs were placed 15–26 mm apart on MHA plate. After incubation for 16–18 hours at 35 ±2°C, a flattening of the zone of inhibition of the clindamycin disc adjacent to the erythromycin disc (referred to as D-zone), or presence of hazy growth within the zone of inhibition around clindamycin even without the classical D-zone, was reported as “clindamycin resistance”.

All Enterococcus spp. isolated were tested for vancomycin resistance using E-test (AB, Biodisk, Solna, Sweden), manufacturer’s instructions were strictly adhered to. The plate was incubated at 37°C for 24 hours. Minimum Inhibitory Concentration (MIC) result was interpreted according to CLSI guideline [16]. Isolates with MIC breakpoint of >16μg/ml were considered resistant to vancomycin resistant.

### Data analysis

Data was entered into Epi Info software version 3.4(CDC, Atlanta GA, USA). The analysis was done with Statistical Package for Social Sciences (SPSS) software version 19.0(SPSS Inc., Chicago, IL., USA). Categorical variables were compared using Pearson’s Chi-square test or Fisher’s exact test. P-values < 0.05 were considered significant for all tests. Mutivariate logistic regression analysis was employed to determine the independent contribution of clinical variables to the prediction of acquisition of ICU infections in the hospital as dependent variables. The same statistical test was used to determine independent predictors of ICU mortality except that APACHE II-adjusted model was used. Variables that had a value of P≤ 0.2 on univariate analysis were entered into a forward stepwise logistic regression model. Goodness-of-fit was evaluated by Hosmer-Lemeshow test. Two tailed p values were reported. The association between the independent determinants of ICU-acquired infections and hospital mortality were estimated using odds ratios and 95% confidence intervals. For outcome analysis, patients were distributed into two subgroups according to survival status (died or discharged).

## Results

During the study period, 139 patients were admitted into the ICU, but only 71 patients were eligible for the study. Among the 68 patients that were excluded, 39 were admitted for less than 48 hours in ICU, 23 were children under the age of 15 years and 6 declined. The male female ratio was 1:0.8 with an age range of 15 years to 69 years, with a mean of 38.7 (±14.9). Length of stay (LOS) in the ICU ranged from 2 to 38 days with a median of 5 and Interquartile range (IQR) of 5–12 days. All patients had urinary catheters inserted for urine output monitoring and 35 (49.3%) had Central venous catheter (CVC) in-situ. Thirty-seven (52.1%) of the patients were either transferred from the ward or another hospital while 34 (47.9%) came via the Accident and Emergency unit (Table 1). Early-onset infections (infection developing between two and seven days on ICU admission) were described for 25 (78.1%) infections, while 7 (21.9%) had late-onset infections.

**Table 1:** Baseline demographic and Clinical characteristics of Patients admitted in the Intensive Care Unit of Lagos University Teaching Hospital, Lagos.

Variables	n	frequency	Mean (SD)	Median (IQR)
Gender				
Female	32	45.1		
Male	39	54.9		
Age	71		38.7(±14.9)	
15–40	43	60.6		
41–69	28	39.4		
Apache II Score	71		21.2 (± 8.07)	
<20	35	49.3		
≥20	36	50.7		
Length of ICU stay	71		9.1 (± 7.3)	7 (5–12)
2–7	41	57.7		
8–38	30	42.3		
Duration of antibiotic administration in ICU (days)	69		8.0 (±6.4)	
Locations before ICU admissions				
Hospital	37	52.1		
Home	34	47.9		
Wards admitted in hospital before transfer to ICU				
Outside hospital	20	28.2		
Accident and Emergency	34	47.9		
Surgical ward	8	11.3		
Medical ward	3	4.2		
Obstetrics and Gynaecology	3	4.2		
Neuro-ward	3	4.2		

### Clinical characteristics of patients

**Fig 1:**
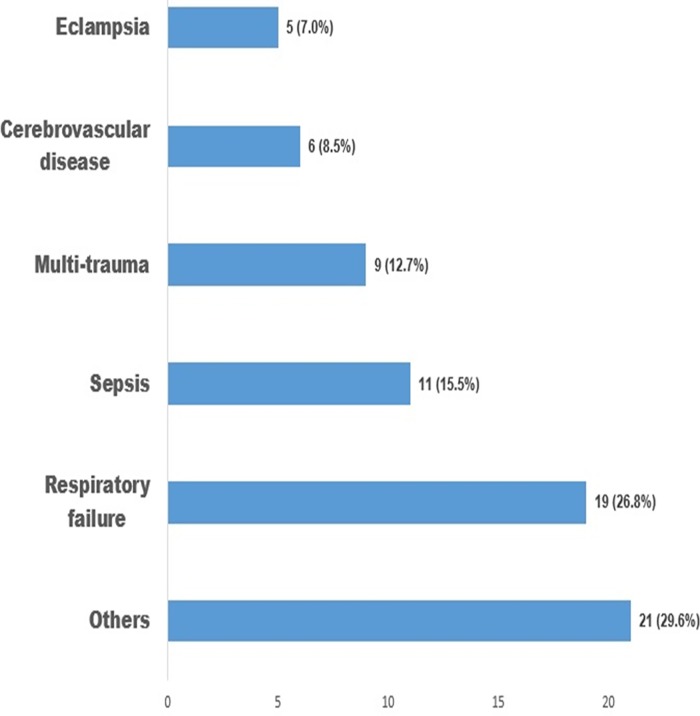
Admitting diagnosis of Patients in Intensive Care Unit of Lagos University Teaching Hospital, Lagos. “Others” as a group of admitting diagnosis included: post-surgical patients, patients with chronic renal failure and malignancies.

Thirty-five (49.3%) of the patients had an APACHE II score of less than 20 while 36 (50.7%) had a score of 20 and above, the mean APACHE II score was 21.2 (±8.07). Admission diagnoses were respiratory failure 19 (26.8%); severe sepsis 11(15.5%); eclampsia 5(7.0%) and others 21 (29.6%) and these included post-surgical patients, patients with chronic renal failure and malignancies) (Fig 1).

**Fig 2:**
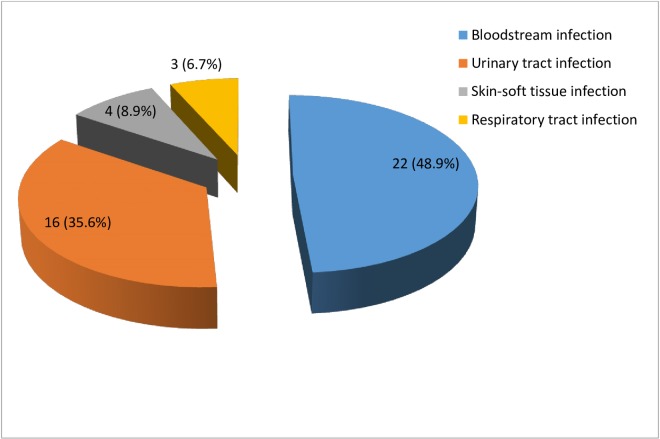
Distribution of Intensive Care Unit-acquired infections in Lagos University Teaching Hospital, Lagos. Bloodstream infection (48.9%); Urinary tract infection (35.6%); Skin-soft tissue infection (8.9%); Respiratory tract infection (6.7%).

A total of 45 laboratory-confirmed infections were identified in 32 patients, representing a prevalence rate of 45.1%, and an incidence rate of 79/1000 patient-days (Incidence rate was derived by dividing the number of new nosocomial infections acquired in a period by Total number of patient-days for the same period x 1000). The total patient-days in the study was 405. The most common infection were bloodstream infections accounting for 49.0% (22/45) of all infections. Two patients had 3 episodes and 3patients had 2 episodes of BSI which were catheter associated. Eleven patients had a total of 16 (35.6%) urinary tract infections (UTI); 1 patient had 3 episodes and 3 patients had 2 episodes each of UTI. A total of 4 (8.9%) skin-soft tissue infections (SSTI) were reported amongst 3 patients, one had 2 episodes of SSTI. Three (6.7%) RTIs were observed in three patients (Fig 2). Of these three, two were on ventilator, while one was not.

### Microbiological profile of pathogens identified

**Fig 3:**
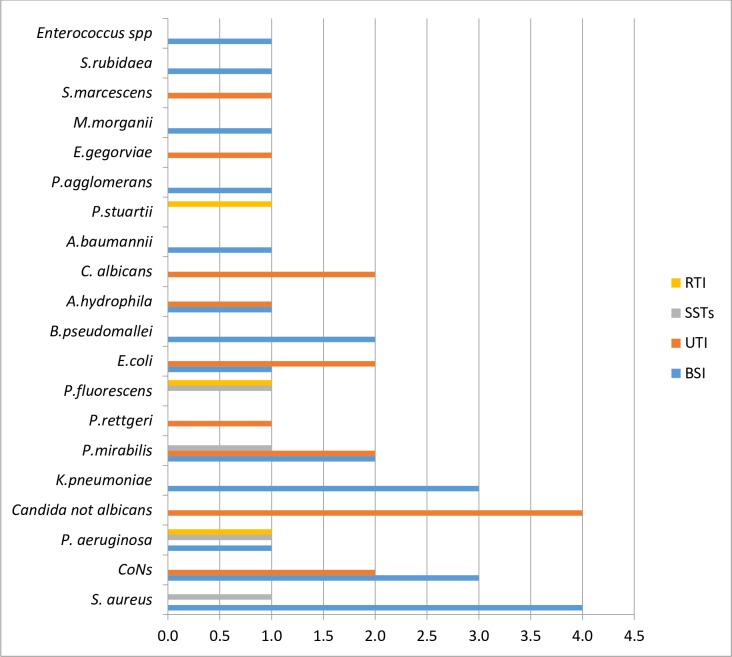
Distribution of Causative microorganisms by the sites of Intensive Care Unit–acquired infections in Lagos University Teaching Hospital, Lagos. KEY: RTI = Respiratory tract infections; SSTs = Skin-soft tissue infections; UTI = Urinary tract infections; BSI = Blood stream infections; CoNS = Coagulase negative staphylococcus.

Twenty different species of pathogenic microorganisms were identified in the 45 infections recorded. *Proteus mirabilis*, *Staphylococcus aureus*, and Coagulase-negative Staphylococci were the most frequently isolated pathogens accounting for 5(11.1%) each respectively. *Staphylococcus aureus* was the most common cause of bloodstream infection, accounting for 4 (18.2%) cases, followed by Coagulase-negative staphylococcus and *Klebsiella pneumoniae* with 3 (13.6%) isolates each. Non-Candida albicans 4 (25.0%) and Coagulase-negative staphylococci (12.5%) were the commonest cause of urinary tract infections (Fig 3).

Eleven gram-positive bacteria were isolated, *S*.*aureus* 5(45.5%), Coagulase negative staphylococcus 5(45.5%), and one *Enterococcus feacalis*. Four (80%) of the *Staph aureus* were Methicillin resistant staphylococci (zones of inhibition were <21mm), Only one (20%) of S. aureus (MRSA) isolates showed resistance to clindamycin, gentamicin, and levofloxacin respectively. However, all *S*. *aureus* isolates were susceptible to ciprofloxacin. Four (80%) of the Coagulase-negative staphylococci were Methicillin resistant (MRCoNS); three (3) were resistant to clindamycin and gentamicin respectively, one displayed resistance to ciprofloxacin but none to levofloxacin. The only *Enterococcus Feacalis*. isolated was vancomycin resistant (VRE) breakpoint of >16μg/ml was used. The resistance profile of the gram negative bacteria revealed that 16(57.1%) were MDR (Table 2).

**Table 2:** Resistance profile of Gram negative isolates from Intensive Care Unit infections in Lagos University Teaching Hospital, Lagos.

Resistance to antibiotics (R/S for single isolates; %R for n ≥ 2 isolates)									
Isolates	n	SAM	FEP	CRO	CIP	GEN	LVX	MEM	TZP
*Proteus mirabilis*	5	60	0	60	100	60	20	0	60
*Escherichia coli*	3	33.3	66.7	66.7	66.7	66.7	33.3	0	100
*Pseudomonas aeruginosa*	3	33.3	0	100	66.7	66.7	33.3	0	0
*Klebsiella pneumoniae*	3	NA	0	100	100	100	100	0	100
*Aeromonas hydrophila*	2	100	0	100	50	50	0	50	50
*Pseudomonas fluorescens*	2	100	0	50	0	100	0	0	100
*Burkholderia pseudomallei*	2	50	50	100	100	100	100	0	50
*Acinetobacter baumannii*	1	R	S	S	S	R	S	S	R
*Pantoea agglomerans*	1	R	R	R	R	R	R	S	R
*Enterobacter gergoviae*	1	R	R	R	R	R	R	S	R
*Morganella morganii*	1	S	S	S	S	S	S	S	S
*Proteus rettgeri*	1	R	S	R	R	R	S	S	S
*Providentia stuartii*	1	R	S	R	R	R	R	S	S
*Serratia rubidaea*	1	R	R	R	R	R	S	R	R
*Serratia marcescens*	1	R	R	R	R	R	S	R	S

KEY: SAM = Ampicillin-sulbactam; FEM = Cefepime; CRO = Ceftriaxone; CIP = Ciprofloxacin; GEN = Gentamicin; LVX = Levofloxacin; MEM = Meropenem; TZP = Piperacillin-tazobactam; R = Resistance; S = Sensitive; NA = Not applicable

### Risk factors and outcome of HAIs in the ICU

Five factors were identified as statistically significant regarding HAIs in the ICU using univariate analysis: Use of antibiotics one month before ICU admission (OR = 0.334; p = 0.03),; surgery one month before admission (OR = 0.181, p< 0.001); urethral catheterization (OR = 5.38; p<0.05), endotracheal intubation (OR = 5.78; p< 0.05), and patients’ location before ICU admission (OR = 0.11; p< 0.05) (Table 3).

**Table 3:** Univariate analysis of risk factors for Intensive Care Unit-acquired infections in Lagos University Teaching Hospital, Lagos.

Factors		Infected n(%)	Not Infected n(%)	Odds ratio	p-value
Use of antibiotic one month before hospital admission				0.334	0.03
• Yes	20(62.5)		14(35.9)		
• No	12(37.5)		25(64.1)		
Surgery one month before admission				0.181	0.001
• Yes	21 (65.6)		(25.6)		
• No	11 (34.4)		29 (74.4)		
Urethral catheterization				5.38	0.03
• Yes	32(100.0)		33(84.6)		
• No	0(0.0)		6(15.4)		
Endotracheal intubation				5.78	0.02
• Yes	29(90.6)		13(33.3)		
• No	3(9.4)		26(66.7)		
Location before admission				0.11	0.001
• Hospital wards	13 (59.1)		4 (13.8)		
• Accident/Emergency unit	9 (40.9)		25 (86.2)		
Age(years)				3.28	0.17
• ≥60	2 (6.25)		(17.9)		
• <59	30 (93.75)		32 (82.1)		
Gender				0.44	0.086
• Female	18 (56.25)		(35.9)		
• Male	14 (43.75)		25 (64.1)		
Malignancy				0.46	0.191
• Yes	9 (28.13)		(15.38)		
• No	23 (71.87)		33 (84.62)		
Length of ICU stay				0.44	0.093
• ≥ 7	17 (53.1)		(33.3)		
• < 7	15 (46.9)		26 (66.7)		
APACHE II score				0.52	0.186
• ≥ 20	19 (59.4)		(43.6)		
• < 20	13 (40.6)		22 (56.4)		

*P*-value < 0.05

Septiceamic patients had higher mortality rates than non-septiceamic patients (75.0% vs 25.0%). Five (5) factors were significantly associated with this severe outcome. These factors were ICU-acquired infection (OR = 8.2; p = 0.04); endotracheal intubation (OR = 5.7; p = 0.04); urethral catheterization (OR = 7.5; p = 0.04); acquisition of infection within seven days of admission (OR = 4.9; p = 0.05) and an APACHE 11 score value greater or equal to 20 (OR = 9.04; P <0000) (Table 4). After controlling for the effect of the APACHE II score on the clinical outcome of infections in ICU, using a forward stepwise multivariate logistic regression method, only ICU-acquired infection was found to be statistically significant for ICU mortality (Table 4).

**Table 4:** Result of Univariate and Multivariate regression analysis of risk factors for ICU mortality.

Univariate analysis			Multivariate analysis		
Risk factors	OR	p-value	OR	CI	p-value
ICU-aquired infection	8.2	0.004	3.83	1.082–13.522	0.04
LOS < 7days before infection	4.9	0.05	0.65	0.190–2.208	0.49
Endotracheal intubation	5.7	0.004	2.7	0.576–13.165	0.21
Urethral catheterization	7.5	0.004	1.79	0.107–29.857	0.69
APACHE II score	9.04	0.000	-	-	-
Location before admission	1.9	0.17	2.2	0.708–6.501	0.18
Age	1.7	0.28			
Gender	0.63	0.32			
Use of antibiotic one month before admission	0.77	0.58			
Surgery one month before admission	0.50	0.24			
Malignancy	1.5	1.00			
Nasogastric intubation	0.97	0.97			
LOS > 7 days	0.81	0.66			

KEY: LOS = Length of ICU stay; OR = Odds ratio; CI = Confidence interval

[-2loglikelihood 77.170; *p*(Hosmer & Lemeshow test) = 0.88] only for the Multivariate analysis

## Discussion

The 45% prevalence rate of HAIs reported is very high, it demonstrates the inadequacy of the infection control processes in place in our ICU. This rate is significantly higher than the pooled prevalence of 35.2% from ICUs in LMICs [2]. A number of reasons could be attributable for this including but not limited to; lack of an antibiotic stewardship program in Centre of study [2,18], lack of trained ICU nurses [19,20, 21], frequent turnover of ICU nurses, poor nurses to patient ratio [20,21], no established infection control policy [2], poor hand hygiene due to irregular water supply in centre of study, high bed occupancy rates, patients relatives are required to pay from pocket for every item used in managing these patients, high level of human traffic in ICU (relatives, students, HCWs) etc. Antibiotic stewardship programs aim to optimize appropriate antibiotic treatment while minimizing antibiotic resistance thus improving patient safety, a recent review by Ramsamy et al (2016) emphasized this [22].

BSIs (49.0%) were the most documented infections in our ICU and this is contrary to previous findings [23,24] which documented hospital acquired pneumonia as the most common type of HAIs in ICU settings. This finding can easily explained by the fact that BAL samples which is a more representative sample are not routinely collected in centre of study and also there was only one functional ventilator during the study period so fewer patients developed ventilator associated pneumoniae. Also in the index ICU, use of a pair of sterile gloves was the best of barrier precautions practiced during catheterization. The common practice of using femoral veins for central venous catheterization instead of subclavian vein could have contributed to this high BSI rate, as there are increased associated infections risks with the later [25]. Due to challenges with financial constraints and need for access for intravenous drugs, most times patients were treated with antibiotics instead of changing cathetres. CA-UTIs at 35.6% were also high and the readily apparent reason is that all patients are catheterized for monitoring. In the ICU studied, the local (institutional) protocol was to change urinary catheter (latex) within 5-7days however we found that catheters were in place for more than 10 days in some patients due to financial constraints. Candida spp., were the most prevalent agents of urinary tract infection in our study and this is probably due to poor catheter care which allows this organism which is part of skin flora to colonize the catheter and eventually become pathogenic following migration into the bladder [26,27]. This finding compares well with that of Rosenthal et al. [27] and that of a more recent study [28].

Intensive care unit (ICU)-acquired infections are a challenging health problem worldwide, especially when caused by multidrug-resistant (MDR) pathogens. Twenty different species of microorganisms were involved in 45 episodes of ICU infections in this study. While Staphylococcus aureus, coagulase-negative Staphylococcus (CoNS), and Proteus mirabilis were the most frequently isolated organisms causing ICU-acquired infections. MDR gram negative bacteria were responsible for about two-thirds of the total infections recorded. This trend contrasts with findings from studies done in developed countries where the prevalent cause of health care-associated infections is switching over to gram positive organisms [29,30]. This findings are however comparable to another study where Enterobacteriaceae, Staphylococcus aureus, and CoNs were the most common isolates associated with BSI [27]. The increase in BSIs due to CoNS in our study is consistent with a report by Hidron and colleagues [31]. The reasons for this new trend seem not readily available, however, epidemiological variables and changes in risk factors (e.g., increased use of invasive procedures in ICU especially presence of CVC lines insitu) may offer plausible explanations.

The most worrisome finding in this study was that almost all the gram positive bacterial agents of BSIs were MDR, including the only Enterococcus spp. Previous studies have reported steady rise of resistant pathogens from patients in ICU [32,33]. The degree of antimicrobial resistance among key pathogens in our hospital’s ICU as revealed by this study was striking, though, a previous similar study conducted in ICUs in developing countries revealed similar high antibiotic resistance rates [26]. Wide use of broad-spectrum antibiotics, prolonged administration of antibiotics, extremely sick patients, and cross-transmission of pathogens via hands and materials, especially where there is low nurse-to-patient ratio as we had in the index ICU, may all have contributed to the high antibiotic resistance rates in this study [34,35].

The highest rates of antibiotic resistance displayed by the bacterial strains were to amoxicillin-clavulanate and ceftriaxone. During the time of the study, these two drugs were the commonest drugs given empirically to ICU patients. This practice might have built up selective pressure that had led to the evolution of resistant strains which clonally expanded over time. This is indeed a notable finding, which addresses one of the key objectives of this study that is: determining the microbiological profile of the index ICU to guide in developing an antibiotic policy for it. A recent ‘surviving sepsis’ publication by Ramsamy et al (2016) noted that ‘unnecessary administration of antimicrobial therapy not only impacts on the individual patient but also on those patients in the same ICU environment’ and that ‘.knowledge of inherent flora and their antimicrobial susceptibility patterns are crucial’ [22].

Our findings revealed a high rate of multi-drug resistant (MDR) gram-negative bacilli (57.1%). A similar study demonstrated comparable high rates of MDR (51%) in gram-negative bacilli among patients in Afghanistan [36]. Discordantly, much lower MDR rates in gram-negative bacilli—5.9% in P. aeruginosa, 1.2% in E. coli and 0.9% in K. pneumonia—were obtained in ICU in Canada [37]. Explanation for this very high rate of MDR (57.1%) with gram-negative bacteria is not clear especially when none of the species was positive for ESBL-production, as most MDR gram-negative bacterial strains are ESBL-producers [38]. However, lack of antibiotic stewardship programs resulting in irrational antibiotic use in developing countries such as Nigeria when compared to Canada can be might account for this variation. Another plausible explanation could be that those gram-negative bacteria expressed both ESBLs and AmpC-like beta-lactamases. The AmpC beta-lactamase-producing strains do not demonstrate lowering of the MIC when combined with clavulanic acid and so are positive on the initial screen test, but negative on the phenotypic confirmation test. So, if such gram-negative strains are tested, AmpC beta-lactamase-producing effect will not allow phenotypic expression of the ESBLs [39]. Unfortunately, we did not test for AmpC beta-lactamase-production in this study.Another significant finding was the high rate (80.0%) of methicillin resistance among the Staphylococcus spp, which is close to the 84.0% prevalence rate obtained by Rosenthal and colleagues [26].

Otherstudies have independently associated acquisition of MDR-ICU-acquired infections with risk factors such as use of antibiotics one month prior to ICU admission, surgery one month before admission, urethral catheterization and endotracheal intubation [40,41]. In our study, only the use of antibiotics one month before ICU admission was independently associated with acquisition of ICU infections and by extension, other multidrug resistant organisms. Contrary to previous observation [40,41], being severely ill (assessed by APACHE II score), and prolonged length of ICU stay seemed not to be risk factors for ICU-acquired infections (MDR by extension) among patients analysed in this study.

Limitations of this study includes relatively small size of study population which is a reflection of the limited ICU beds available and the fact that healthcare generally is out of pocket in the study environment. We speculate that this could have contributed to the study’s lack of power to detect some significant relationships from our data. Secondly, the convenience sampling technique we used helped us to access our study participants easily, however, it could have introduced sampling bias, distorting good representation of the entire population.

In conclusion, ICU-acquired infections remained a significant risk factor for ICU- mortality even after adjusting for APACHE II score. There is dire need to develop and entrench an antibiotic stewardship policy in our ICU and set up a national surveillance program to monitor infections in our Nigeria. A robust infection control program is also a matter of urgency in our setting.

## Supporting Information

S1 File Proforma. (DOCX)

S2 File Ethical Clearance. (JPG)

S3 File Consent form. (DOCX)

S4 File APACHE II Score. (DOCX)

S5 File Laboratory Procedures. (DOCX)
