# Supplementary material for: Incidence, Clinical Outcome and Risk Factors of Intensive Care Unit Infections in the Lagos University Teaching Hospital (LUTH), Lagos, Nigeria
Source: PLoS One. 2016 Oct 24;11(10):e0165242. doi: 10.1371/journal.pone.0165242 (PMC5077115; doi:10.1371/journal.pone.0165242)
Supplement: S1 File — (DOCX) [file pone.0165242.s001.docx]

## **PROFORMA**

### **Intensive Care Unit Infections in Lagos University Teaching Hospital,**

### **Idi-Araba**

**PATIENT’S DETAILS:**

Study number

Hosp number

Date of birth

Age (years)

Gender: male female

**CLINICAL DETAILS:**

Date of admission

Indication (diagnosis) for admission

Location before ICU admission Home Hospital

If referred from hospital; which ward in the hospital?

Infectious disease type diagnosed in ICU

Site of infection in ICU

Source of culture Blood CSF Urine Other (specify)

Date of specimen collection

Causative agent of the diagnosed infectious disease

Length of days in ICU before infections was diagnosed

**ANTIMICROBIAL DETAILS:**

Prior use of antibiotic one month before admission No Yes

If yes; names amikacin cefotaxime ceftriaxone

Ceftazidime cefepime cefuroxime

Ciprofloxacin cloxacillin co-amoxiclav

Meropenem trimethoprim-sulphamethoxazole

Any other (specify)

Route of administration oral parenteral

Duration of administration (days)

Antibiotic administration during ICU admission No Yes

If yes; names amikacin cefotaxime ceftriaxone

Ceftazidime cefepime cefuroxime

Ciprofloxacin cloxacillin co-amoxiclav

Meropenem trimethoprim-sulphamethoxazole

Any other (specify)

Route of administration oral parenteral

Duration of administration (days)

Other underlying diseases (specify)

Assessment of underlying disease (APACHE* II) score

Possible risk factors

- Immunosuppression Yes No
- Surgery Yes No
- Chronic renal failure Yes No
- Chronic lung disease Yes No
- Malignancy Yes No
- Neutropenia Yes No
- Dialysis Yes No
- Diabetes mellitus Yes No
- Long term steroid use Yes No
- Endotracheal tube use Yes No
- Central venous catheters Yes No
- Urethral catheters use Yes No
- Tracheostomy Yes No
- Inadequate hand hygiene Yes No
- Nasogastric tube Yes No

**OUTCOME DETAILS:**

Total duration of ICU stay (days)

Duration of ICU stay after acquisition of ICU infection (days):

≤5 ≤ 10 ≤ 15 20 ≤

Outcome: discharged died

Date of death or discharge from ICU

*Acute Physiological and Chronic Health Evaluation.
