# Supplementary figures and images for: Incidence, Clinical Outcome and Risk Factors of Intensive Care Unit Infections in the Lagos University Teaching Hospital (LUTH), Lagos, Nigeria
Source: PLoS One. 2016 Oct 24;11(10):e0165242. doi: 10.1371/journal.pone.0165242 (PMC5077115; doi:10.1371/journal.pone.0165242)

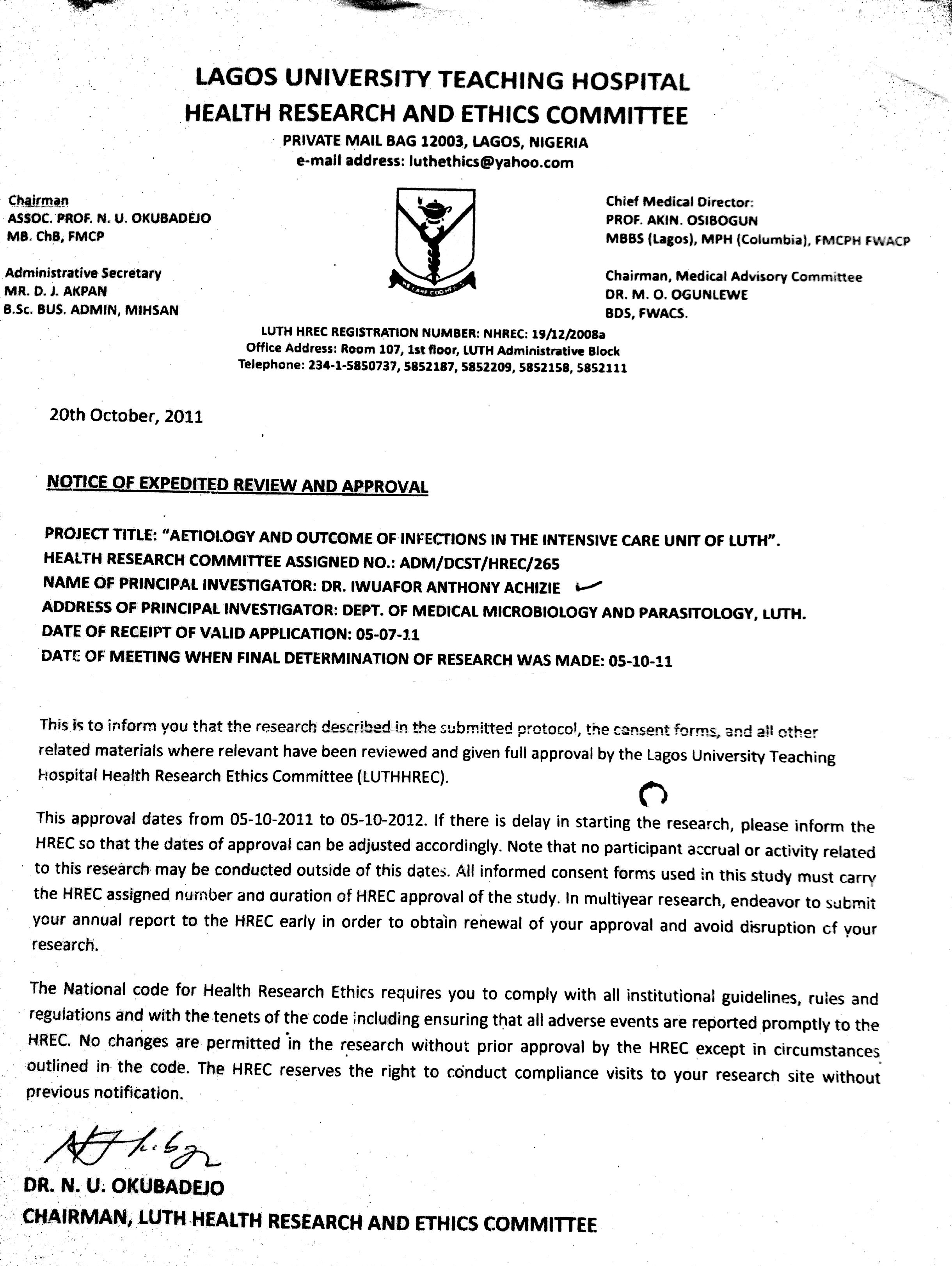

Supplement: S2 File — (JPG) [file pone.0165242.s002.jpg]
