# Supplementary material for: Incidence, Clinical Outcome and Risk Factors of Intensive Care Unit Infections in the Lagos University Teaching Hospital (LUTH), Lagos, Nigeria
Source: PLoS One. 2016 Oct 24;11(10):e0165242. doi: 10.1371/journal.pone.0165242 (PMC5077115; doi:10.1371/journal.pone.0165242)
Supplement: S3 File — (DOCX) [file pone.0165242.s003.docx]

## CONSENT FORM

**Date / / /**

### **Intensive Care Unit Infections in Lagos University Teaching Hospital, Lagos**

Contents of the Consent form was explained to the patients in the language they best understand.

My name is Dr. Anthony Iwuafor of the Department of Medical Microbiology and Parasitology, Lagos University Teaching Hospital, Idi-Araba.I am carrying out the above-mentioned research topic (approved by the Research and Ethics Committee of LUTH) to investigate the causes of infections in the Intensive Care Unit of the Hospital, and also how these infections affect the admitted patients in terms of hospital-stay and recovery.

During this exercise, I will require you to tell me why you came to the hospital and the drugs you are taking. Blood samples (5-10mls), urine, wound swab, and/or sputum may be taken from you, first day on admission and 48 hours later. The process of taking the specimen will not cause you any extra pain or injury as the tests will constitute routine investigations needed for your management. More so, you will not be required to pay for the tests.

The information you give shall be kept very confidential and shall be useful to the Department of Medical Microbiology and Parasitology and to improve the management of patients with similar conditions. You are free to decline to participate in this study. You have the right to withdraw at any given time if you choose to, and the quality of care you are receiving will not be compromised in any way. If you have any questions concerning this research study, please call me at 08033441539.

Sincerely,

**Dr. Anthony A Iwuafor.**

**……………………………………….. ........../........./..........**

**Signature Date**

**I agree to take part in this Project.**

**.................................................... ......../......../............**

**Patient’s/Relative Signature Date**

**................................................... ........./.........../...........**

**Witness Signature Date**
