# Supplementary material for: Incidence, Clinical Outcome and Risk Factors of Intensive Care Unit Infections in the Lagos University Teaching Hospital (LUTH), Lagos, Nigeria
Source: PLoS One. 2016 Oct 24;11(10):e0165242. doi: 10.1371/journal.pone.0165242 (PMC5077115; doi:10.1371/journal.pone.0165242)
Supplement: S4 File — (DOCX) [file pone.0165242.s004.docx]

## **ACUTE PHYSIOLOGICAL AND CHRONIC HEALTH EVALUATION (APACHE II) SCORE**

1. Age (years)

<44 0

45-54 2

55-64 3

65-74 5

>74 6

2. History of severe organ insufficiency or immunocompromised?

Yes, and non-operative or emergency post-operative patient 5

Yes, and elective post-operative patient 2

No 0

3. Temperature (Celsius)

>40.9 4

39-40.9 3

38.5-38.9 1

36-38.4 0

34-35.9 1

32-33.9 2

30-31.9 3

<30 4

4. Mean arterial pressure (mmHg)

>159 4

130-159 3

110-129 2

70-109 0

50-69 2

<50 4

5. Heart rate

>179 4

140-179 3

110-139 2

70-109 0

55-69 2

40-54 3

<40 4

6. Respiratory rate (Non-ventilated or ventilated)

>49 4

35-49 3

25-34 1

12-24 0

10-11 1

6-9 2

<6 4

7. Oxygenation (Use PaO_2_ if FiO_2_ <50%, otherwise use A-a gradient)

A-a grad >499 4

A-a grad 350-499 3

A-a grad 200-349 2

A-a grad <200 (if FiO_2_ >49%) or PO_2_ >70 (if FiO_2_ <50%) 0

pO_2_ =61-70 1

pO_2_ =55-60 3

pO_2_ <55 4

8. Arterial pH

>7.69 4

7.60-7.69 3

7.50-7.59 1

7.33-7.49 0

7.25-7.32 2

7.15-7.24 3

<7.15 4

9. Serum Sodium (mMol/L)

>179 4

160-179 3

155-159 2

150-154 1

130-149 0

120-129 2

111-119 3

<111 4

10. Serum Potassium (mMol/L)

>6.9 4

6-6.9 3

5.5-5.9 1

3.5-5.4 0

3-3.4 1

2.5-2.9 2

<2.5 4

11. Serum Creatinine (mg/100mL)

>3.4 and Acute renal failure 8

2-3.4 and Acute renal failure 6

>3.4 and Chronic 4

1.5-1.9 and Acute renal failure 4

2-3.4 and Chronic 3

1.5-1.9 and Chronic 2

0.6-1.4 0

<0.6 2

12. Haematocrit (%)

>59.9 4

50-59.9 2

46-49.9 1

30-45.9 0

20-29.9 2

<20 4

13. White blood count (Total/mm^3^ in 1000’s)

>39.9 4

20-39.9 2

15-19.9 1

3-14.4 0

1-2.9 2

<1.0 4

14. 15 minus Glasgow coma scale ( )

**TOTAL SCORE _______________________________________________**
