# Supplementary material for: Incidence, Clinical Outcome and Risk Factors of Intensive Care Unit Infections in the Lagos University Teaching Hospital (LUTH), Lagos, Nigeria
Source: PLoS One. 2016 Oct 24;11(10):e0165242. doi: 10.1371/journal.pone.0165242 (PMC5077115; doi:10.1371/journal.pone.0165242)
Supplement: S5 File — (DOCX) [file pone.0165242.s005.docx]

## **LABORATORY PROCEDURES**

**Catalase Test**

The catalase test is most commonly used to differentiate members of the *Micrococcaceae* from members of the *Streptococcaceae.* Materials needed are 3% hydrogen peroxide, an 18- to 24-hour test organism, positive control (*Staphylococcus aureus)* and negative control (*Streptococcus species*). Procedure: With an inoculating needle, growth from the centre of a colony will be transferred to the surface of a glass slide. One drop of 3% hydrogen peroxide will be added and then observed for bubble formation. Result interpretation: Rapid and sustained appearance of bubbles or effervescence indicate a positive test.

**Coagulase Test**

The coagulase test is used to identify *Staphylococcus aureus* and differentiate it from most other species of *Staphylococci.* The coagulase is present in two forms, bound and free, each having different properties that require the use of separate testing procedure. Materials required: rabbit plasma with EDTA, a known *Staphylococcus aureus* strain and a *Staphylococcus epidermidis* strain as positive and negative controls, respectively. Procedure: 1. Slide test (bound coagulase): two drops of sterile normal saline (in two circles drawn with wax pencil) will be dropped on a glass slide. The colony material from the organism will be gently emulsified in liquid in each of the circles. A drop of plasma will be placed in the suspension in one of the circles and mixed with a wooden applicator stick. To the other suspension, a drop of saline will be placed as control. The slide will be rocked back and forth while observing for agglutination of the test suspension. 2. Tube test (free coagulase): A small amount of the colony growth of the organism will be emulsified in a tube containing 0.5 mL of plasma. The tube will be incubated at 35°C for 4 hours and observed for clot formation by gently tilting the tube. If no clot is observed at that time, the tube will be re-incubated at room temperature and read again after 18 hours. Result interpretation: 1. Slide test: a positive reaction will be detected within 10 to 15 seconds of mixing the plasma with the suspension by the formation of white precipitate and agglutination of the organisms in the suspension. The test will be considered negative if no agglutination is observed after 2 minutes. All strains that are coagulase-positive will be reported as coagulase-positive *Staphylococcus* or less precisely, *Staphylococcus aureus*. All strains producing negative slide tests will be tested with the tube coagulase test. 2. *Tube test*: this will be considered positive if any degree of clotting is noted. The tube will be gently tilted and not agitated, because this may disrupt partially formed clotted material.

**PYR Test:**

This is a rapid test for the presumptive identification of both group A β-haemolytic *Streptococci* and *Enterococci.* The materials needed for this test include: PYR broth (Todd-Hewitt broth with 0.01% L-naphthylamide-β-naphthylamide) dispensed into sterile tubes in 0.20-mL volumes, PYR reagent (0.01% *p*-dimethylaminocinnamaldehyde), positive control (*Enterococcus faecalis* or *Streptococcus pyogenes)* and negative control (*Streptococcus agalactiae*). Procedure: growth of two or three morphologically similar colonies will be picked with sterile wire loop and these will be emulsified in the small volumes of PYR broth. The tube will be incubated at 35ºC for 4 hours, and then one drop of PYR agent will be added and observed for colour change. The reaction will be read and recorded one minute after the addition of the reagent. Result interpretation: the development of a deep cherry red colour within a minute of addition of the reagent will be interpreted as positive while a yellow or orange colour will be considered negative.

**Optochin Susceptibility Test:**

Ethylhydrocupreine hydrochloride (optochin), a derivative of quinine, selectively inhibits the growth of *Streptococcus pneumoniae* at very low concentrations (5 µg/mL or less). Materials needed will include colonies of the organism to be tested on sheep blood agar, sheep blood agar plate, optochin discs (5 µg), positive control (*Streptococcus pneumoniae*), negative control (Viridans streptococcus or *Enterococcus faecalis).* Procedure: using a sterile wire loop, three to four colonies of the organism to be tested will be selected and streaked onto one-third of a blood agar plate. An optochin disc will then be placed in the upper third of the streaked area. The plate will be incubated at 35ºC for 18 to 24 hours in a candle jar or in 5-10%. Result interpretation: a Viridans streptococcus will be presumptively identified as *S. pneumonia*e if it shows a zone of inhibition of 14 mm or more around a 6-mm (oxoid) disc. Organisms showing zones smaller than these should be tested for bile solubility.

**Bile Solubility Test:**

Bile salts, specifically sodium deoxycholate and sodium taurocholate, have the capability to lyse *Streptococcus pneumoniae* selectively when added to actively growing bacteria in agar or broth media. Materials needed will include a pure culture of the test organism grown at 35ºC for 18 to 24 hours on Sheep blood agar plate, 2% sodium deoxycholate, positive (bile-soluble) control (*Streptococcus pneumoniae*), negative (bile-insoluble) control (Viridans streptococcus). Procedure: to well-isolated colonies of the test organism growing on sheep blood agar, a drop of 2% sodium deoxycholate will be added, without inverting the plate, it will be placed in a 35ºC incubator for 30 minutes. Result interpretation: bile-soluble colonies on which the reagent was placed disappear, leaving a partially haemolyzed area where the colony had been, while the bile-insoluble (negative reaction) colonies where the reagent was placed remain intact and visible.

**Bile-Aesculin Test:**

The test is based on the ability of certain bacteria, for example, the group D streptococci and *Enterococcus* species to hydrolyze aesculin in the presence of bile (4% bile salt or 40% bile). Materials needed will include bile-aesculin agar medium, positive control (*Enterococcus* species e.g., *E. faecalis*), negative control (Viridans streptococcus). Procedure: with a sterile wire loop, two or three morphologically similar streptococcal colonies will be touched and used to inoculate slant of bile aesculin medium or plate. The tube will be incubated at 35ºC for 24-48 hours in an ambient air incubator. Result interpretation: diffuse blackening of more than half of the slant within 24-48 hours indicates aesculin hydrolysis. All group D streptococci will be bile-aesculin positive within 48 hours.
